# Avacincaptad pegol for geographic atrophy secondary to age-related macular degeneration: 18-month findings from the GATHER1 trial

**DOI:** 10.1038/s41433-023-02497-w

**Published:** 2023-03-24

**Authors:** Sunil S. Patel, David R. Lally, Jason Hsu, Charles C. Wykoff, David Eichenbaum, Jeffrey S. Heier, Glenn J. Jaffe, Keith Westby, Dhaval Desai, Liansheng Zhu, Arshad M. Khanani

**Affiliations:** 1 West Texas Retina Consultants, Abilene, TX USA; 2 New England Retina Consultants, Springfield, MA USA; 3 The Retina Service of Wills Eye Hospital, Wills Eye Physicians-Mid Atlantic Retina, Philadelphia, PA USA; 4 Retina Consultants of Texas, Houston, TX USA; 5 Retina Vitreous Associates of Florida, St. Petersburg, FL USA; 6 Morsani College of Medicine at The University of South Florida, Tampa, FL USA; 7 Ophthalmic Consultants of Boston, Boston, MA USA; 8 Department of Ophthalmology, Duke University, Durham, NC USA; 9 IVERIC Bio, Inc, New York, NY USA; 10 Sierra Eye Associates and The University of Nevada, Reno School of Medicine, Reno, NV USA

**Keywords:** Retinal diseases, Outcomes research

## Abstract

**Background/Objectives:**

To assess the safety and efficacy of avacincaptad pegol (ACP), a C5 inhibitor, for geographic atrophy (GA) secondary to age-related macular degeneration (AMD) over an 18-month treatment course.

**Subjects/Methods:**

This study was an international, prospective, randomized, double-masked, sham-controlled, phase 2/3 clinical trial that consisted of 2 parts. In part 1, 77 participants were randomized 1:1:1 to receive monthly intravitreal injections of ACP 1 mg, ACP 2 mg, or sham. In part 2, 209 participants were randomized 1:2:2 to receive monthly ACP 2 mg, ACP 4 mg, or sham. The mean rate of change of GA over 18 months was measured by fundus autofluorescence.

**Results:**

Compared with their respective sham cohorts, monthly ACP treatment reduced the mean GA growth (square root transformation) over 18 months by 28.1% (0.168 mm, 95% CI [0.066, 0.271]) for the 2 mg cohort and 30.0% (0.167 mm, 95% CI [0.062, 0.273]) for the 4 mg cohort. ACP treatment was generally well tolerated over 18 months, with most ocular adverse events (AEs) related to the injection procedure. Macular neovascularization (MNV) was more frequent in both 2 mg (11.9%) and 4 mg (15.7%) cohorts than their respective sham control groups (2.7% and 2.4%).

**Conclusions:**

Over this 18-month study, ACP 2 mg and 4 mg showed continued reductions in the progression of GA growth compared to sham and continued to be generally well tolerated. A pivotal phase 3 GATHER2 trial is currently underway to support the efficacy and safety of ACP as a potential treatment for GA.

## Introduction

Dry age-related macular degeneration (AMD) is characterized in its early stages by a thickening of the Bruch’s membrane and accumulation of deposits on the apical retinal pigment epithelium (RPE) surface (subretinal drusenoid deposits) and on Bruch’s membrane beneath the RPE (drusen) [1]. Geographic atrophy (GA) is the advanced form of dry AMD, identified by irreversible loss (atrophy) of RPE, photoreceptors, and choriocapillaris. While GA lesions often first appear in the nonfoveal region of the macula, progression over time eventually causes them to include the foveal center, leading to irreversible central vision loss [2]. In half of the GA cases, the disease progresses to both eyes within 7 years of initial diagnosis, showcasing the potentially severe impact on quality of life [3]. Despite advancements in the AMD treatment, there is still a large unmet need as GA continues to be a leading cause of central vision loss with no approved therapies [4].

Evidence suggests a role of the complement pathway in AMD and GA, and the potential for complement system components as therapeutic targets [5–7]. C3, C5b-9, CFB, and CFH have been detected in drusen [8], and elevated plasma levels of C3a, C3d, and C5a have been observed in AMD patients [9–11]. Cleavage of C3 and C5 are the converging points of the various pathways within the complement cascade, making these enzymes ideal targets for therapeutic inhibition.

While an overactive complement pathway is implicated in several pathological processes, the complement system also plays an important role in the maintenance of retinal integrity by eliminating immune complexes and apoptotic cells and mediating adaptive immune functions [12]. Preclinical studies showed that C3aR- and C5aR-mediated signaling was necessary to maintain normal retinal function and structure [13]. Therefore, an approach targeting the cascade downstream could be preferable, inhibiting deleterious consequences of complement overactivation while preserving the beneficial properties of the complement system. While C3 inhibition is wide-ranging and affects multiple complement pathways, the effects of C5 inhibition are restricted to C5a and the membrane attack complex (MAC), preserving some C3 activity [14]. Accordingly, findings from preclinical models suggest inhibition of C3 as a therapeutic strategy in AMD may be detrimental in the long term [15], and that the genetic ablation of C3 in mice decreases apoptotic cell clearance and accelerates photoreceptor degeneration [16].

GATHER1 was a recent international, prospective, randomized, double-masked, sham-controlled, pivotal phase 2/3 clinical trial [17]. During GATHER1, avacincaptad pegol (ACP) (IVERIC bio, Inc., Parsippany, NJ), an inhibitor of C5 cleavage, was assessed in participants with GA and showed promising results [17]. For the primary endpoint assessment at 12 months, there was a significant reduction of GA growth (square root transformation) in both the 2 mg (27.4%, *p* = 0.0072) and 4 mg (27.8%, *p* = 0.0051) treatment cohorts compared to their respective sham cohorts, with an acceptable safety profile.

In this paper, we present the final report covering the entire 18-month treatment period of GATHER1, which is noteworthy for the following 3 reasons. First, to continue to demonstrate an acceptable safety profile. Second, to assess a persistent efficacy by means of separation of GA growth rates between treatment and sham cohorts. Third, to evaluate the potential of the drug to prevent consequent central vision loss, which usually happens after 12 months of follow-up [18].

## Methods

### Study design

We have previously reported the detailed study design for GATHER1 (NCT02686658) [17]. Briefly, GATHER1 was an international, prospective, randomized, double-masked, sham-controlled, phase 2/3 pivotal clinical trial that enrolled 286 participants with GA at 63 sites (United States, Europe, and Israel) between January 2016 and October 2018. GATHER1 consisted of two simultaneous parts. In part 1, eyes were randomized 1:1:1 to receive ACP 1 mg, ACP 2 mg, or sham. In part 2, eyes were randomized 1:2:2 to receive ACP 2 mg (one 100 µl injection + 1 sham administration), ACP 4 mg (two 2 mg injections, total volume 200 µl), or sham (2 sham administrations). Investigators administered monthly intravitreal (IVT) injections of ACP or sham for a duration of 18 months. Eyes were randomized and stratified for baseline GA lesion area, best-corrected visual acuity (BCVA), and pattern of fundus autofluorescence (FAF). Participants, investigators, independent reading center personnel (Duke Reading Center, Duke University, Durham, NC), and sponsor personnel were masked to the treatment that eyes received during the study. GATHER1 was performed in accordance with the tenets of the Declaration of Helsinki and the International Conference on Harmonisation Good Clinical Practice guidelines. The appropriate ethics committee or institutional review board at each study center approved the protocol. Informed consent was obtained from all participants. All data were collected in compliance with the Health Insurance Portability and Accountability Act and other applicable laws. An Independent Data Monitoring Committee reviewed participant safety data during the trial.

### Enrollment criteria

Candidates with GA ≥ 50 years of age with BCVA between 20/25 and 20/320 in the study eye were eligible for enrollment. The selection of the study eye was based on the ophthalmic inclusion and exclusion criteria. In cases where both eyes of a candidate were eligible, the choice of the study eye was at the investigator’s discretion. The GA lesion had to be non–center point involving, and, in part, within 1.5 mm from the foveal center. Total lesion area had to be between 2.5 and 17.5 mm^2^, determined by screening FAF images. For multifocal lesions, at least 1 lesion had to be 1.25 mm^2^ or larger. The total GA area had to be able to be photographed in its entirety within a 30-degree photographic field centered on the foveal center.

Candidates were excluded if they had macular atrophy secondary to any condition other than AMD in either eye (e.g., myopic degeneration or hereditary retinal degeneration). Candidates must not have received any prior treatment for AMD or any prior IVT treatment for any indication in either eye, apart from oral vitamins or mineral supplements. The proposed study eye was also excluded if any subtype of macular neovascularization (MNV) was detected in either eye, or if any ocular condition was present in the study eye that could progress during the study and potentially affect central vision or otherwise act as a confounding factor, or if there was any sign of diabetic retinopathy in either eye.

### Image acquisition

Blue light FAF, fundus photographic, and fluorescein angiographic images were obtained with the modified 3-field protocol (field 1 M: 30-degree field centered on the temporal optic nerve border; field 2: 30-degree field centered on the foveal center; and field 3 M: 30-degree field centered 1 to 1.5 disc diameters temporal to the center of field 2). Heidelberg Spectralis or HRA systems (Heidelberg Engineering, Heidelberg, Germany) were used to capture FAF and near-infrared (IR) field 2 images. Heidelberg Spectralis or Cirrus (Carl Zeiss Meditec, Jena, Germany) systems were used to acquire spectral-domain optical coherence tomography (OCT) scans. Spectralis scans were obtained with 97-line volume scan (20° × 20°, high-resolution mode, Automatic Real-Time [ART] = 9) and 73-line volume scan (20° × 15°, high-resolution mode, ART = 9) protocols. Cirrus OCT scans were obtained with 512 × 128 macular cube and 5-line high-definition raster scan protocols.

### Endpoints

GA lesion area was measured by FAF at baseline, month 6, month 12, and month 18. The prespecified primary efficacy endpoint was the mean change in the GA lesion area at month 12. Independent masked readers (Duke Reading Center) measured the GA area on FAF images using the RegionFinder^TM^ software (Heidelberg Engineering), while OCT and IR images were used to help define GA boundaries. Secondary endpoints included the mean change from baseline in BCVA (Early Treatment Diabetic Retinopathy Study [ETDRS] letters) and low-luminance BCVA (LL-BCVA; ETDRS letters). Safety endpoints included incidence of adverse events (AEs) and serious adverse events (SAEs).

### Statistical analysis

All efficacy analyses were conducted for the intent-to-treat (ITT) population, which consisted of all randomized eyes that received at least 1 dose of study treatment. Comparisons were made between ACP 2 mg and ACP 4 mg with their corresponding sham, as determined in a prespecified statistical analysis plan. The analyses were repeated at month 18 using the same conventions as were prespecified for month 12. It should be emphasized that month 18 analyses were descriptive and not protected from a type I error; hence, no P-values are reported to avoid any misinterpretation of the results. The primary efficacy analysis and 18-month analysis used the mixed-model with repeated measures (MMRM) analysis to compare the treatment arms, which uses all observed data with the assumption of data missing at random. All safety analyses were performed on the safety population, which included all subjects who received at least 1 dose of the study drug. In the event participants received a dose different from the one assigned according to the randomization schedule, safety analyses were conducted according to the dose received rather than according to the dose assigned by randomization. Safety measures were calculated based on observed cases.

## Results

### Participants and baseline characteristics

Overall, 286 eyes with GA of 286 participants were enrolled in this 2-part study. Part 1 randomized 77 eyes: 26 eyes (ACP 1 mg), 25 eyes (ACP 2 mg), and 26 eyes (sham). Part 2 randomized 209 eyes: 42 eyes (ACP 2 mg), 83 eyes (ACP 4 mg), and 84 eyes (sham). Baseline characteristics were balanced among cohorts (Table S1). In total, 201 participants completed the study through the total treatment period (Table S2). At baseline FAF images, diffuse-trickling pattern was observed in 18.2% of study eyes in the 2 mg cohort vs. 18.3% in the associated sham cohort, and 15.9% in the 4 mg cohort vs. 20.5% in the associated sham cohort.

### 18-month efficacy endpoint analysis

**Fig. 1:**
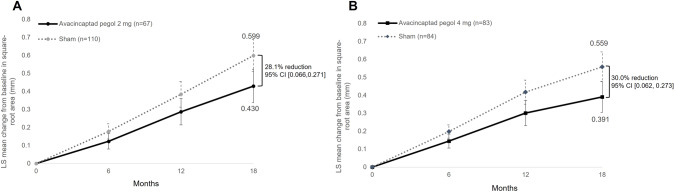
Mean change of baseline GA lesion size over 18 months (least squares mean). **A** 2 mg avacincaptad pegol compared to sham (**B**) 4 mg avacincaptad pegol compared to sham.

Since the primary analysis at 12 months, the observation at month 18 showed a further separation of the treatment arm vs. sham over the additional 6-month period for both ACP doses. GA lesion growth, square root transformed, was reduced by 28.1% (0.168 mm, 95% CI [0.066, 0.271]) in the ACP 2 mg–treated eyes when compared to sham (least squares mean values of 0.60 and 0.43 mm, respectively). Likewise, GA lesion growth was reduced by 30.0% (0.167 mm, 95% CI [0.062, 0.273]) in ACP 4 mg–treated eyes when compared to sham (least squares mean values of 0.56 and 0.39 mm, respectively) (Fig. 1 and Table 1). Data analysis using non–square root transformation also revealed reductions in GA lesion growth rates with ACP over the 18-month period: 32.2% (1.156 mm^2^, 95% CI [0.480, 1.833]) with ACP 2 mg vs. sham (2.431 and 3.587 mm^2^, respectively) and by 29.4% (1.029 mm^2^, 95% CI [0.345, 1.708]) with ACP 4 mg vs. sham (2.460 and 3.486 mm^2^, respectively) (Tables S3 and S4). A piecewise linear slope analysis per time period is presented in Table S4.

**Table 1:** Mean rate of change in GA area from baseline by month, mixed-model for repeated measures (MMRM) analysis square root transformation.

Cohort	Avacincaptad pegol 2 mg	Sham^a^	Avacincaptad pegol 4 mg	Sham^b^
GA at baseline (mm), Mean (SD)	2.618 (0.7001)	2.633 (0.7009)	2.715 (0.7320)	2.636 (0.7091)
*n* at baseline	67	110	83	84
GA at month 6 (mm), Mean (SD)	2.772 (0.7183)	2.865 (0.7154)	2.930 (0.7483)	2.886 (0.7145)
6-month least squares Mean (SE)	0.123 (0.043)	0.178 (0.042)	0.145 (0.039)	0.198 (0.037)
6-month difference, (% Difference [95% CI])		0.054 (30.68 [0.006; 0.103])		0.053 (26.93 [0.007; 0.100])
*n* at month 6 (% of baseline)	58 (86.6)	92 (83.6)	63 (75.9)	73 (86.9)
GA at month 12 (mm), Mean (SD)	3.032 (0.6643)	3.119 (0.7182)	3.083 (0.7713)	3.143 (0.7201)
12-month least squares Mean (SE)	0.287 (0.073)	0.384 (0.071)	0.301 (0.070)	0.418 (0.067)
12-month difference, (% Difference [95% CI])		0.097 (25.31 [0.017; 0.177])		0.117 (28.04 [0.033; 0.202])
*n* at Month 12 (% of baseline)	49 (73.1)	90 (81.8)	55 (66.3)	72 (85.7)
GA at month 18 (mm), Mean (SD)	3.063 (0.5821)	3.282 (0.7563)	3.108 (0.8158)	3.294 (0.7641)
18-month least squares Mean (SE)	0.430 (0.092)	0.599 (0.089)	0.391 (0.087)	0.559 (0.083)
18-month difference, (% Difference [95% CI])		0.168 (28.11 [0.066; 0.271])		0.167 (29.97 [0.062; 0.273])
*n* at Month 18 (% of baseline)	41 (61.2)	78 (70.9)	45 (54.2)	64 (76.2)

*GA* geographic atrophy, *SD* standard deviation, *SE* standard error.

^a^Sham for 2 mg arm.

^b^Sham for 4 mg arm.

As expected, an overall decline in visual acuity was observed, except for the LLVA change in the ACP 4 mg cohort and corresponding sham. Participants receiving ACP experienced numerically better outcomes in BCVA or LLVA over 18 months when compared with their corresponding sham groups. Specifically, the mean (SE) change in BCVA (ETDRS letters) from baseline to month 18 was −12.7 (4.29) for the ACP 2 mg cohort vs. −15.1 (4.12) for the corresponding sham (2.37 difference); and −4.27 (4.24) for the ACP 4 mg cohort vs. −7.07 (4.06) for the corresponding sham (2.8 difference). The LLVA change from baseline to month 18 was −2.72 (4.29) for the ACP 2 mg cohort vs. −3.10 (4.03) for the corresponding sham (0.37 difference); and 2.85 (3.86) for the ACP 4 mg cohort vs. 1.68 (3.70) for the corresponding sham (1.17 difference) (Table 2).

**Table 2:** Mean change in best-corrected visual acuity and low-luminance best-corrected visual acuity (ETDRS letters) from baseline to month 18.

Cohort	Avacincaptad pegol 2 mg	Sham^a^	Difference
*n* at baseline	67	110	
Baseline BCVA, Mean (SE)	70.2 (10.01)	69.0 (10.35)	1.2
n at month 18, (% of baseline)	48 (71.6)	83 (75.5)	
Change in BCVA, Mean (SE)	−12.7 (4.29)	−15.1 (4.12)	2.37
Baseline LLVA, Mean (SE)	36.7 (21.10)	34.5 (19.32)	2.2
Change in LLVA, Mean (SE)	−2.72 (4.21)	−3.10 (4.03)	0.37

Cohort	Avacincaptad pegol 4 mg	Sham^b^	Difference
*n* at baseline	83	84	
Baseline BCVA, Mean (SE)	69.5 (9.81)	68.3 (11.03)	1.2
*n* at month 18, (% of baseline)	47 (56.6)	65 (77.4)	
Change in BCVA, Mean (SE)	−4.27 (4.24)	−7.07 (4.06)	2.80
Baseline LLVA, Mean (SE)	36.8 (20.87)	33.9 (18.77)	2.9
Change in LLVA, Mean (SE)	2.85 (3.86)	1.68 (3.70)	1.17

*BCVA* best-corrected visual acuity, *GA* geographic atrophy, *LLVA* low-luminance visual acuity, *SE* standard error.

^a^Sham for 2 mg arm.

^b^Sham for 4 mg arm.

### Safety analysis

Intravitreal ACP was generally well tolerated over the 18-month period, with no ocular or systemic treatment-emergent AEs (TEAEs) related to the study drug. The major safety data are reported in Table 3. There was 1 ocular SAE (1.5%; optic ischemic neuropathy) with ACP 2 mg and 1 (1.2%; retinal detachment) with ACP 4 mg. The most frequently reported ocular AEs were related to the injection procedure. AEs occurring in ≥2% of the eyes in either the 2 mg, 4 mg, or sham cohorts are summarized in Table S5. One eye (1.5%) in the 2 mg ACP cohort developed intraocular inflammation (IOI) during the study, a case of vitritis, at month 7 without any anterior chamber inflammation. This event was mild with no effect on visual acuity, no treatment was given, and injections of the study drug proceeded as scheduled with complete resolution of the IOI by month 11. Per the investigator, the event was not drug- or injection procedure-related. No cases of endophthalmitis were reported with ACP or sham. There were only 3 total ocular TEAEs in the ACP treatment groups leading to study drug discontinuation over the 18-month period, none related to the study drug as per the investigators. The TEAEs were cystoid macular degeneration (4 mg, 1.2%), ischemic optic neuropathy (2 mg, 1.5%), and retinal detachment (4 mg, 1.2%).

**Table 3:** Major reported safety data from study patient population over 18 months.

AEs, No. of participants (%)	Avacincaptad pegol 2 mg (*n* = 67)	Avacincaptad pegol 4 mg (*n* = 83)	Sham (*n* = 110)
Ocular SAEs, study eye^a^	1 (1.5)	1 (1.2)	0
Ocular SAEs, fellow eye	0	0	0
Ocular TEAEs, study eye	39 (58.2)	61 (73.5)	45 (40.9)
Ocular TEAEs, fellow eye	15 (22.4)	24 (28.9)	25 (22.7)
Study drug-related ocular TEAEs, study eye	0	0	0
Endophthalmitis	0	0	0
Intraocular inflammation^a^	1	0	0
Discontinuation from trial related to study drug	0	0	0
Systemic SAEs	11 (16.4)	20 (24.1)	28 (25.5)
Study drug-related systemic SAEs	0	0	0
Systemic TEAEs	41 (61.2)	53 (63.9)	66 (60.0)
Study drug-related systemic TEAEs	0	0	0

*AE* adverse event, *SAE* serious adverse event, *TEAE* treatment-emergent adverse event.

^a^Not drug-related per investigators.

### Macular neovascularization conversion

The term MNV was used to refer to each of 3 types of neovascularization: type 1 when the neovascularization is located external to the RPE, type 2 when internal to the RPE (subretinal), and type 3 when arising within the neurosensory retina (retinal angiomatous proliferation) [19]. The investigators reported MNV using 2 MedDRA Preferred Terms (PT): “choroidal neovascularization” (CNV) and “neovascular age-related macular degeneration” (nvAMD). Therefore, these 2 MedDRA PTs were summed to provide an accurate incidence of MNV. Macular neovascularization conversion rates were 11.9% with ACP 2 mg and 15.7% with ACP 4 mg, compared to 2.7% and 2.4% in their respective sham groups (Table 4). Fellow-eye conversion rates were 3.0%, 3.6%, and 3.6% for ACP 2 mg, 4 mg, and sham, respectively. When the trial was designed, it was assumed that the development of MNV would prevent accurate FAF measurements, and therefore all study eyes that developed MNV were exited from the study.

**Table 4:** Incidence of macular neovascularization in the study eye through 18 month.

Number of participants (%)	Avacincaptad pegol 2 mg (*n* = 67)	Avacincaptad pegol 4 mg (*n* = 84)	Sham (*n* = 110)
nAMD^a^	8 (11.9)	9 (10.7)	2 (1.8)
CNV^a^	0	4 (4.8)	1 (0.9)
Combined	**8 (11.9)**	**13 (15.7)**	**3 (2.7)**

The bolded values are the summation of the two rows above.

*nAMD* neovascular age-related macular degeneration, *CNV* choroidal neovascularization.

^a^The terms “nAMD” and “CNV” were the MeDRA preferred terms used by investigators to describe macular neovascularization.

## Discussion

This report presents the results for one of the longest treatment periods with a complement inhibitor of eyes with GA. The data are consistent with the 12-month results previously published, and further support ACP as a potential treatment to reduce GA lesion growth [17]. The study showed 28.1% and 30.0% mean reductions in GA lesion growth over the 18-month study for the 2 mg and 4 mg treatment cohorts, respectively, with continued separation from their respective sham cohorts when compared to the 12-month time point. Aligned with these results, other studies also continue to support the complement system as an attractive target for GA therapies [20]. In this study, the mean change in GA lesion area was reported as a square root transformation with an MMRM analysis to alleviate the effect of baseline lesion size on GA growth rate [21, 22]. Additionally, we reported GA lesion growth, untransformed, as has been traditionally reported [3, 23]. Similar to the 12-month data, there were no pronounced differences between a square root and non–square root transformation analysis.

The visual disability related to GA is often underestimated because BCVA is generally a poor indicator of functional vision in these patients, as the fovea may be spared until late disease stages [24]. The enlarging scotoma may have a more significant effect in earlier disease stages, limiting activities such as reading and driving. Based on historical data, significant vision loss does not occur, on average, until past the 18-month time frame when GA progression generally enters the central fovea [24]. Even so, visual acuity is not an ideal surrogate for foveal center point involvement. For GA lesions that abut the foveal center point at baseline, patients often adapt and fixate eccentrically, and further progression may not have a noticeable effect on BCVA. Also, the extent of foveal encroachment can result in varying degrees of central vision loss. For a lesion that just barely progresses into the fovea, the visual acuity impact might not be noticeable, while if the GA extends not just to the center point, but significantly beyond, the effect on visual acuity could be substantially greater. The observed reductions in GA area growth in this study could possibly delay AMD-related functional vision loss and central visual acuity loss, thereby possibly prolonging the quality of life for GA patients [2]. Following these patients over an extended period could provide valuable insights and will be the subject of a future post hoc analysis.

While 3 intravitreal dosage regimens were evaluated (1, 2, and 4 mg), the 12- and 18-month reports focused on the 2 and 4 mg study groups. The efficacy outcomes in the 2 and 4 mg ACP study groups were similar, with only an additional 1.9% reduced GA growth with the higher dose. However, the incidence of AEs and AEs related to the injection procedure was higher with the 4 mg dose, most likely due to the increased volume of the injection (200 μl vs. 100 μl) and not necessarily the study drug dosage. Considering the similar efficacy and better safety profile, the 2 mg dose was chosen for a subsequent confirmatory phase 3 study (GATHER2, NCT04435366).

Intravitreal ACP over 18 months was generally well tolerated, with no cases of endophthalmitis and only one mild episode of IOI (vitritis) that did not result in study discontinuation. Recent investigations of intravitreal complement inhibitors for GA have revealed increased rates of study eye MNV conversion and, similar to the results at month 12 in GATHER1, increased MNV conversion was observed with 2 mg (11.9%) and 4 mg (15.7%) ACP at 18 months. The results include all investigator-determined MNV subtypes, with or without exudation [19]. Unfortunately, since the eyes that developed MNV exited the study, clinical details on the MNV course and the impact on BCVA are limited. Fellow-eye MNV conversion rates were 3.0%, 3.6%, and 3.6% for the ACP 2 mg, 4 mg, and sham groups, respectively. These rates are lower than previously reported rates of fellow-eye MNV conversion, estimated between 6% and 12% per year [25, 26]. Some possible explanations for this discrepancy could be the absence of reading center confirmation for fellow eyes, or that investigators might have been more focused on examining the study eye, failing to identify a subclinical MNV in the fellow eye.

The reasons behind the increased rates of MNV in eyes with GA receiving investigational complement inhibitors are not known. Perhaps the clinical efficacy of the study drugs retains healthier vascular endothelial growth factor A (VEGF-A)–producing cells that lead to increased rates of MNV conversion compared to the control group [27]. Another possibility is that by inhibiting the production of C3a and C5a, a switch from proinflammatory M1 macrophages to pro-angiogenic M2 macrophages may occur. A consequent pro-angiogenic milieu in the retina would either lead to higher rates of MNV or the onset of exudation of previously quiescent lesions [28]. In an experimental model of laser-induced CNV, mice lacking either C3 or C5 showed increased neovascularization compared to controls [29]. A third possibility is that inflammasome activation in microglial cells and macrophages may release cytokines that help maintain homeostasis of the choroidal vasculature and mitigate VEGF production in eyes with AMD, thereby decreasing the risk of MNV [30]. However, inflammasome activation in immune cells may lead to RPE degeneration, increasing the risk of GA [31]. By inhibiting the inflammasome formation, complement inhibition may therefore be slowing down RPE degeneration while simultaneously permitting increased VEGF production. Lastly, the regulator of cell cycle gene (RGCC), a gene that responds to complement activation and induces apoptosis in endothelial cells, is highly and specifically expressed in the choriocapillaris. In fact, RGCC was the most upregulated choriocapillaris gene in a donor diagnosed with AMD [32]. Blocking the complement pathway can prevent apoptosis and favor the development of MNV. Future studies may want to examine the development and progression of MNV, particularly nonexudative, as they may recapitulate the choriocapillaris, possibly providing better nourishment to the RPE and outer retina. After all, the presence of type I MNV has been associated with a slower progression of the areas of atrophy, suggesting a possible protective effect [33]. Conversely, de novo MNV could also have a detrimental effect on visual acuity, especially if associated with higher flow or exudative/hemorrhagic characteristics. While the design of this study and the number of MNV occurrences limits the ability to confirm or refute any hypothesis, we aim to answer these questions through secondary analyses on additional ongoing trials.

Despite the rigorously controlled study design, some limitations must be acknowledged. First, the macular imaging included color fundus photography, FAF, fluorescein angiography, and OCT but did not include OCT angiography, which has the novel ability to noninvasively detect the presence of MNV and determine its subtype [34, 35]. Second, it has been shown that distinct patterns of abnormally increased FAF in the junctional zone of GA have an impact on GA progression. In particular, the diffuse-trickling pattern has relatively faster GA progression than other diffuse types (3.02 mm^2^/year vs. 1.67 mm^2^/year) [36]. Although the percentages were similar between ACP 2 mg and sham (18.2% vs. 18.3%), there was a difference between ACP 4 mg and sham (15.9% vs. 20.5%) that could have contributed to a faster growth rate in the sham group. Lastly, a limitation inherent to clinical trials is the occurrence of missed visits and participants that exit the study prematurely, which could have potentially introduced a bias in the study. While a seemingly high rate of early discontinuation among the 4 mg cohort (44.6%) was observed, the use of the MMRM model in our statistical analysis attempted to mitigate the impact of missing data and the prespecified sensitivity analyses indicated that it had a small impact on the overall conclusions. Moreover, the discontinuation rate in the 2 mg cohort (28.4%), which was the dose chosen for the subsequent GATHER2 phase 3 trial, was similar to its corresponding sham (26.7%).

In GATHER1, a pivotal phase 2/3 study, there was a consistent reduction in GA growth with monthly intravitreal ACP 2 mg or 4 mg compared to sham through month 18. These findings reinforce the previously published 12-month results and support the ongoing safety and efficacy of complement inhibition in eyes with GA. These results support targeting C5 inhibition within the complement cascade as a promising method to treat GA, and longer-term ACP therapy may potentially provide a cumulative clinical benefit over time. GATHER2, a phase 3 trial, has recently been completed to further investigate the efficacy and safety of ACP 2 mg for GA.

## Summary

### What was known before

- Despite advancements in the AMD treatment, there is still a large unmet need as GA continues to be a leading cause of central vision loss with no approved therapies.
- Evidence suggests a role of the complement pathway in AMD and GA and the potential for complement system components as therapeutic targets during GATHER1;avacincaptad pegol (ACP), an inhibitor of C5 cleavage, showed a significant reduction of GA growth (square root transformation) in both the 2 mg (27.4%, *p* = 0.0072) and 4 mg (27.8%, *p* = 0.0051) treatment cohorts compared to their respective sham cohorts at 12 months.

### What this study adds

- Since the primary analysis at 12 months, the observation at month 18 showed a further separation of the treatment arm vs. sham over the additional 6-month period for both ACP doses.
- GA lesion growth, square root transformed, was reduced by 28.1% (0.168 mm, 95% CI [0.066, 0.271]) with ACP 2 mg vs. sham (least squares mean values of 0.60 and 0.43 mm, respectively), and by 30.0% (0.167 mm, 95% CI [0.062, 0.273]) with ACP 4 mg vs. sham (least squares mean values of 0.56 and 0.39 mm, respectively).
- Intravitreal ACP over 18 months was generally well tolerated, with no cases of endophthalmitis and only one mild episode of IOI (vitritis) that did not result in study discontinuation.
- MNV conversion rates were 11.9% with ACP 2 mg and 15.7% with ACP 4 mg, compared to 2.7% and 2.4% in their respective sham groups.

### Supplementary information

Supplemental Table 1

Supplemental Table 2

Supplemental Table 3

Supplemental Table 4

Supplemental Table 5

## Data Availability

The data that support the findings of this study are available on reasonable request from IVERIC bio, Inc. but restrictions may apply to their availability due to their containing information that could compromise the privacy of research participants.
