# Correction: Avacincaptad pegol for geographic atrophy secondary to age-related macular degeneration: 18-month findings from the GATHER1 trial

**DOI:** 10.1038/s41433-023-02548-2

**Published:** 2023-05-26

**Authors:** Sunil S. Patel, David R. Lally, Jason Hsu, Charles C. Wykoff, David Eichenbaum, Jeffrey S. Heier, Glenn J. Jaffe, Keith Westby, Dhaval Desai, Liansheng Zhu, Arshad M. Khanani

**Affiliations:** 1 West Texas Retina Consultants, Abilene, TX USA; 2 New England Retina Consultants, Springfield, MA USA; 3 The Retina Service of Wills Eye Hospital, Wills Eye Physicians-Mid Atlantic Retina, Philadelphia, PA USA; 4 Retina Consultants of Texas, Houston, TX USA; 5 Retina Vitreous Associates of Florida, St. Petersburg, FL USA; 6 Morsani College of Medicine at The University of South Florida, Tampa, FL USA; 7 Ophthalmic Consultants of Boston, Boston, MA USA; 8 Department of Ophthalmology, Duke University, Durham, NC USA; 9 IVERIC Bio, Inc, New York, NY USA; 10 Sierra Eye Associates and The University of Nevada, Reno School of Medicine, Reno, NV USA

**Keywords:** Retinal diseases, Outcomes research

Correction to: *Eye* 10.1038/s41433-023-02497-w

In the sub-section “Safety analysis,” the percentage 0.6% in the sentence “There was 1 ocular SAE (0.6%; optic ischemic neuropathy) with ACP 2 mg and 1 (1.2%; retinal detachment) with ACP 4 mg” was corrected to 1.5%. In the same section, the percentage in the sentence “One eye (0.6%) in the 2 mg ACP cohort developed intraocular inflammation (IOI) during the study, a case of vitritis, at month 7 without any anterior chamber inflammation” was again corrected to 1.5%. Furthermore, the sentence “The TEAEs were cystoid macular degeneration (4 mg, 1.2%), ischemic optic neuropathy (2 mg, 2.4%), and retinal detachment (4 mg, 1.2%).” was corrected to read “The TEAEs were cystoid macular degeneration (4 mg, 1.2%), ischemic optic neuropathy (2 mg, 1.5%), and retinal detachment (4 mg, 1.2%)”. Finally, the footnote of Supplementary Table 4 was corrected from “Based on a mixed-effects model for repeated measures assuming a piecewise linear trend in time with knots at Month 6, Month 12, and Month 18” to “Based on a mixed-effects model for repeated measures assuming a piecewise linear trend in between times: 0 to Month 6, Month 6 to Month 12, and Month 12 to Month 18”. The original article has been corrected.

