# Supplementary material for: Avacincaptad pegol for geographic atrophy secondary to age-related macular degeneration: 18-month findings from the GATHER1 trial
Source: Eye (Lond). 2023 Mar 24;37(17):3551–7. doi: 10.1038/s41433-023-02497-w (PMC10686386; doi:10.1038/s41433-023-02497-w)
Supplement: Supplementary file 1 — Supplemental Table 1 [file 41433_2023_2497_MOESM1_ESM.pdf]

Supplemental Table 1: Demographics and Baseline Characteristics of the Intent-to-Treat Participants

Cohort	Avacincaptad Pegol 2 mg n = 67	Sham* n = 110	Avacincaptad Pegol 4 mg n = 83	Sham† n = 84
**Mean Age, Years**	78.8	78.2	79.2	78.2
**Female Gender, Number (%)**	45 (67.2)	79 (71.8)	58 (69.9)	61 (72.6)
**Active Smoker, Number (%)**	25 (37.3)	36 (32.7)	26 (31.3)	29 (34.5)
**Noncenterpoint involving GA‡, Number (%)**	62 (92.5)	104 (94.5)	81 (97.6)	82 (97.6)
**Mean GA Area, mm^2**	7.33	7.42	7.90	7.45
**Mean SQ Root GA Area, mm**	2.62	2.63	2.72	2.64
**Bilateral GA, Number (%)**	67 (100)	108 (98.2)	83 (100)	83 (98.8)
**Hyper FAF (%)**	66 (98.5)	109 (99.1)	82 (98.8)	83 (98.8)
**Trickling Pattern, Number (%)**	12 (18.2)	20 (18.3)	13 (15.9)	17 (20.5)
**Mean BCVA (ETDRS Letters)**	70.2	69.0	69.5	68.3
**Mean LL-BCVA (ETDRS Letters)**	36.7	34.5	36.8	33.9
**LL-VD (ETDRS Letters)**	33.5	34.5	32.7	34.4

BCVA = best-corrected visual acuity; GA = geographic atrophy; LL-BCVA = low-luminance best-corrected visual acuity; LL-VD = low-luminance visual acuity deficit (BCVA minus LL-BCVA); SD = standard deviation.
*Sham for 2 mg arm.
†Sham for 4 mg arm.
‡ includes disease in the fovea and outside the fovea but does not involve the foveal center point; refers to observations of baseline images at the Reading Center after participants had been enrolled
