# Supplementary material for: Avacincaptad pegol for geographic atrophy secondary to age-related macular degeneration: 18-month findings from the GATHER1 trial
Source: Eye (Lond). 2023 Mar 24;37(17):3551–7. doi: 10.1038/s41433-023-02497-w (PMC10686386; doi:10.1038/s41433-023-02497-w)
Supplement: Supplementary file 2 — Supplemental Table 2 [file 41433_2023_2497_MOESM2_ESM.pdf]

Supplemental Table 2: Participant disposition through Month 18

Number of participants (%)	Avacincaptad Pegol 2 mg n = 67	Sham* n = 110	Avacincaptad Pegol 4 mg n = 83	Sham† n = 84
**Number of participants who completed through**				
Month 3	63 (94.0)	104 (94.5)	76 (91.6)	81 (96.4)
Month 6	57 (85.1)	99 (90.0)	68 (81.9)	78 (92.9)
Month 9	55 (82.1)	95 (86.4)	62 (74.7)	74 (88.1)
Month 12	53 (79.1)	91 (82.7)	56 (67.5)	72 (85.7)
Month 15	50 (74.6)	88 (80.0)	50 (60.2)	70 (83.3)
Month 18	48 (71.6)	85 (77.3)	46 (55.4)	68 (81.0)
Early discontinuations prior to Month 18	19 (28.4)	25 (22.7)	37 (44.6)	16 (19.0)
Adverse event	1 (1.5)	2 (1.8)	2 (2.4)	1 (1.2)
Protocol violation	0	0	0	0
Investigator decision	1 (1.5)	1 (0.9)	2 (2.4)	1 (1.2)
Sponsor decision	7 (10.4)	3 (2.7)	13 (15.7)	2 (2.4)
Withdrawal by participant	9 (13.4)	12 (10.9)	17 (20.5)	8 (9.5)
Lost to follow-up	0	3 (2.7)	0	1 (1.2)
Participant non-compliance	0	1 (0.9)	0	1 (1.2)
Death	1 (1.5)	1 (0.9)	1 (1.2)	1 (1.2)
Other	0	2 (1.8)	2 (2.4)	1 (1.2)

*Sham for 2 mg arm.

†Sham for 4 mg arm.
