# Supplementary material for: Avacincaptad pegol for geographic atrophy secondary to age-related macular degeneration: 18-month findings from the GATHER1 trial
Source: Eye (Lond). 2023 Mar 24;37(17):3551–7. doi: 10.1038/s41433-023-02497-w (PMC10686386; doi:10.1038/s41433-023-02497-w)
Supplement: Supplementary file 3 — Supplemental Table 3 [file 41433_2023_2497_MOESM3_ESM.pdf]

Supplemental Table 3: Mean Rate of Change in GA Area From Baseline by Month, Mixed-Model for Repeated Measures (MMRM) Analysis, Non-Square Root Transformation

Cohort	Avacincaptad pegol 2 mg	Sham*	Avacincaptad pegol 4 mg	Sham†
**n at Baseline**	67	110	83	84
**GA at Baseline (mm^2), Mean (SD)**	7.335 (3.7930)	7.419 (3.8384)	7.902 (4.1793)	7.446 (3.8934)
**GA at Month 6 (mm^2), Mean (SD)**	8.188 (4.1261)	8.717 (4.2305)	9.138 (4.6019)	8.833 (4.2504)
**6-Month Least Squares Mean (SE)**	0.738 (0.228)	1.056 (0.223)	0.928 (0.210)	1.214 (0.199)
**6-Month Difference, (% Difference [95% CI])**		0.319 (30.17 [0.063;0.575])		0.286 (23.54 [0.034;0.538])
**n at Month 6 (% of Baseline)**	58 (86.6)	92 (83.6)	63 (75.9)	73 (86.9)
**GA at Month 12 (mm^2), Mean (SD)**	9.625 (4.2755)	10.237 (4.5332)	10.090 (5.0055)	10.392 (4.5696)
**12-Month Least Squares Mean (SE)**	1.564 (0.451)	2.181 (0.438)	1.924 (0.428)	2.610 (0.408)
**12-Month Difference, (% Difference [95% CI])**		0.617 (28.29 [0.123;1.111])		0.686 (26.28 [0.171;1.200])
**n at Month 12 (% of Baseline)**	49 (73.1)	90 (81.8)	55 (66.3)	72 (85.7)
**GA at Month 18 (mm^2), Mean (SD)**	9.710 (3.7553)	11.339 (5.0421)	10.309 (5.4363)	11.424 (5.0778)
**18-Month Least Squares Mean (SE)**	2.431 (0.609)	3.587 (0.588)	2.460 (0.565)	3.486 (0.534)
**18-Month Difference, (% Difference [95% CI])**		1.156 (32.24 [0.480;1.833])		1.026 (29.44 [0.345;1.708])
**n at Month 18 (% of Baseline)**	41 (61.2)	78 (70.9)	45 (54.2)	64 (76.2)

GA = geographic atrophy; SD = standard deviation; SE = standard error
*Sham for 2 mg arm.
†Sham for 4 mg arm.
