# Supplementary material for: Avacincaptad pegol for geographic atrophy secondary to age-related macular degeneration: 18-month findings from the GATHER1 trial
Source: Eye (Lond). 2023 Mar 24;37(17):3551–7. doi: 10.1038/s41433-023-02497-w (PMC10686386; doi:10.1038/s41433-023-02497-w)
Supplement: Supplementary file 4 — Supplemental Table 4 [file 41433_2023_2497_MOESM4_ESM.pdf]

Supplemental Table 4: Analysis of Change from Baseline in the GA Lesion Area Measured by FAF of Study Eyes in the ACP 2 mg Cohort Compared to Sham

Time Period	Group	Rate of GA Lesion Area Growth (mm^2)		
		Slope	Difference (95% CI) in slope from Sham	Percent Difference from Sham
**GATHER1 (ACP 2 mg N = 67, Sham N = 110)**				
**Baseline to last month**	ACP 2 mg	2.11	−1.238 (−2.15 to −0.324)	-37.0%
	Sham	3.34		
**Baseline to Month 6**	ACP 2 mg	0.59	−0.299 (−0.545 to −0.052)	-33.6%
	Sham	0.89		
**Month 6 to Month 12**	ACP 2 mg	0.72	−0.401 (−0.731 to −0.071)	-35.9%
	Sham	1.12		
**Month 12 to Month 18**	ACP 2 mg	0.80	−0.538 (−0.874 to −0.201)	-40.2%
	Sham	1.34		

GA, geographic atrophy; FAF, fundus autofluorescence; ACP, avacincaptad pegol; CI, confidence interval
Based on a mixed-effects model for repeated measures assuming a piecewise linear trend in between times: 0 to Month 6, Month 6 to Month 12, and Month 12 to Month 18.
