# Supplementary material for: Avacincaptad pegol for geographic atrophy secondary to age-related macular degeneration: 18-month findings from the GATHER1 trial
Source: Eye (Lond). 2023 Mar 24;37(17):3551–7. doi: 10.1038/s41433-023-02497-w (PMC10686386; doi:10.1038/s41433-023-02497-w)
Supplement: Supplementary file 5 — Supplemental Table 5 [file 41433_2023_2497_MOESM5_ESM.pdf]

Supplemental Table 5: Ocular TEAEs Occurring in ≥ 2% of the Study Population in either the 2 mg or 4 mg Study Cohorts

Number of Participants (%)<br>MedDRA Preferred Term	Avacincaptad pegol 2 mg (n=67)	Avacincaptad pegol 4 mg (n=83)	Sham (n=110)
**Conjunctival hemorrhage**	11 (16.4)	27 (32.5)	13 (11.8)
**Transient injection related events†**	7 (10.4)	22 (26.5)	1 (0.9)
**Choroidal neovascularization***	8 (11.9)	13 (15.7)	3 (2.7)
**Punctate keratitis**	4 (6.0)	6 (7.2)	8 (7.3)
**Conjunctival hyperemia**	3 (4.5)	9 (10.8)	4 (3.6)
**Eye pain**	2 (3.0)	8 (9.6)	3 (2.7)
**Vitreous detachment**	2 (3.0)	4 (4.8)	6 (5.5)
**Conjunctival edema**	2 (3.0)	5 (6.0)	4 (3.6)
**Visual acuity reduction**	3 (4.5)	3 (3.6)	5 (4.5)
**Cataract**	4 (6.0)	2 (2.4)	4 (3.6)
**Eye irritation**	3 (4.5)	2 (2.4)	4 (3.6)
**Vision blurred**	1 (1.5)	3 (3.6)	2 (1.8)
**Corneal Abrasion**	2 (3.0)	1 (1.2)	3 (2.7)
**Lacrimation increased**	2 (3.0)	3 (3.6)	0
**Retinal hemorrhage**	0	3 (3.6)	2 (1.8)
**Eyelid dermatochalasis**	0	2 (2.4)	3 (2.7)
**Dry eye**	0	2 (2.4)	2 (1.8)
**Conjunctivitis**	1 (1.5)	2 (2.4%)	1 (0.9)
**Vitreous floaters**	1 (1.5)	2 (2.4)	1 (0.9)
**Blepharitis**	0	2 (2.4)	1 (0.9)
**Photopsia**	2 (3.0)	1 (1.2)	0
**Viral conjunctivitis**	0	2 (2.4%)	0
**Visual impairment**	0	2 (2.4)	0

*Combines choroidal neovascularization with MedDRA Neovascular AMD.

†Includes increased intraocular pressure and transient retinal artery occlusions; of note 2 mg dose = 100 microliters of total volume, 4 mg = 200 microliters of total volume
